# Circ_MAPK9 promotes STAT3 and LDHA expression by silencing miR-642b-3p and affects the progression of hepatocellular carcinoma

**DOI:** 10.1186/s13062-023-00442-1

**Published:** 2024-01-02

**Authors:** Kunyuan Wang, Qianting Lu, Yufeng Luo, Ganxiang Yu, Zhilei Wang, Jiaen Lin, Zhenlin Tan, Yueqiong Lao, Shiming Liu, Hui Yang

**Affiliations:** 1 Department of Gastroenterology, The Second Affiliated Hospital of Guangzhou Medical University, 250 Changgang East Road, Guangzhou, Guangdong China; 2 Guangzhou Institute of Cardiovascular Disease, The Second Affiliated Hospital of Guangzhou Medical University, Guangzhou, Guangdong China

**Keywords:** Hepatocellular carcinoma, Circ_MAPK9, CeRNA, Proliferation, Metastasis, Aerobic glycolysis

## Abstract

**Background:**

Aberrant expression and activation of circular RNAs (circRNAs) are closely associated with various cancers. The role of circ_MAPK9 (hsa_circ_0001566) in cancer progression remains unknown. This study aims to investigate the function, mechanism and clinical significance of circ_MAPK9 in hepatocellular carcinoma (HCC).

**Methods:**

Circ_MAPK9 expression on the microarray of tumor from clinical HCC patients was detected by in situ hybridization (ISH). Circ_MAPK9 knockdown was achieved with siRNAs in SMMC-7721 and SK-Hep1 HCC cell lines. The biological function of circ_MAPK9 was verified in vitro by CCK8 test, colony formation assay, transwell assay, PI-Annexin V staining, and in vivo by xenograft tumor in nude mice. Fluorescent in situ hybridization (FISH), subcellular fractionation assay, a dual-luciferase reporter assay and rescue experiments were employed for further mechanistic investigation.

**Results:**

The expression of circ_MAPK9 was significantly up-regulated in HCC tissues and cells, which was found to be associated with poor prognosis. Patients with high expression of circ_MAPK9 had a shorter overall survival and disease-free survival in comparison to those with low circ_MAPK9 expression. Functional assays showed that circ_MAPK9 knockdown suppressed cellular proliferation, migration, invasion and tumor growth in vivo, and promoted apoptosis in HCC cells. Moreover, we found that circ_MAPK9 knockdown could inhibit aerobic glycolysis by decreasing the production of adenosine triphosphate (ATP) and lactic acid, which was mediated by lactate dehydrogenase (LDHA). Mechanistically, circ_MAPK9 functioned as ceRNA via sponging miR-642b-3p and alleviated the inhibitory effect of miR-642b-3p on its target signal transducer and activator of transcription 3 (STAT3) and LDHA, thereby leading to STAT3 activation and LDHA expression.

**Conclusions:**

Circ_MAPK9, as an oncogene, promotes HCC growth and metastasis through miR-642b-3p/STAT3-LDHA axis. Circ_MAPK9 could serve as a potential biomarker for HCC poor prognosis and diagnosis.

**Supplementary Information:**

The online version contains supplementary material available at 10.1186/s13062-023-00442-1.

## Introduction

Primary liver cancer is the sixth most common and the fourth most lethal malignancy [1]. The most common type of liver cancer is hepatocellular carcinoma (HCC), which constitutes approximately 90% of liver cancer diagnoses [1], meaning HCC is a major threat to human health worldwide. Despite rapid advances in diagnosis and treatment in recent years, HCC prognosis remains discouraging due to its advanced stage at diagnosis [2].The poor systemic response to advanced HCC is due to the lack of effective and specific biomarkers and/or enrichment strategies [3]. Elucidating the molecular mechanisms of HCC progression facilitates the identification of effective biomarkers for predicting response and patient selection.

Circular RNAs (circRNAs) are a class of novel RNA molecules characterized by covalently closed loops, which make them highly tolerant to exonucleases [4]. Various research groups have screened and identified multiple functional circRNAs associated with HCC [5]. These circRNAs act as oncogenes or tumor suppressors in HCC and influence HCC phenotypes in different ways, including sustained proliferation signaling, activation of invasion and metastasis, angiogenesis and avoidance of cell death [6–8]. The increasing evidences imply that circRNAs regulate the diversity of cellular processes by acting as miRNA sponges, anchors for circRNAs binding proteins (cRBPs), transcriptional regulators, molecular scaffolders, and translation sources of small proteins/peptides [4, 5]. Currently, studies have validated that circRNAs could be used as valuable biomarkers to support the diagnosis of HCC [9]. However, the functions and mechanisms of most annotated circRNAs in HCC remain unclear.

Circ_MAPK9 (also known as hsa_circ_0001566) is located on chr5:179688683–179,707,608 of chromosome and derived from exons of mitogen-activated protein kinase (MAPK9) genome. Previous reports indicated that circ_MAPK9 is highly expressed in peripheral blood mononuclear cells (PBMCs) of patients with rheumatoid arthritis (RA) and promotes RA progression [10, 11]. However, the role of circ_MAPK9 in HCC remains unknown. In this study, we investigated the biological function of circ_MAPK9 in HCC using clinical specimens, transinfected cell experiments, and a mouse xenograft model.

## Materials and methods

### Cell culture and reagents

Human HCC cell lines SMMC-7721 and SK-Hep1 were obtained from the Cell Bank of Type Culture Collection (Chinese Academy of Sciences, Shanghai, China). SMMC-7721 and SK-Hep1 cells were incubated in Dulbecco’s modified Eagle’s medium (DMEM, Thermo Fisher Scientific, Inc., Waltham, MA, United States) supplemented with 10% (V/V) fetal bovine serum (FBS, Gibco) at 37℃ in a humidified incubator containing 5% CO_2_. Recombinant human interleukin 6 (IL-6) was obtained from MULTISCIENCES (LIANKE) BIOTECH, CO., LTD (Hangzhou, China, cat. no. 200-06).

### Cell transfection

Two siRNAs targeting the back-splicing site of circ_MAPK9 and negative control were synthesized by Genepharma (Shanghai, China). MiR-642b-3p mimics, inhibitors and negative control were obtained from Genepharma (Shanghai, China) as well. Full-length of human STAT3 DNA was amplified and subcloned into pcDNA3.1 to establish STAT3 overexpression plasmids (WZ Biosciences Inc, Shandong, China). Lentiviral vector for the complementary oligonucleotides of small hairpin RNA targeting circ_MAPK9 were constructed by HANBIO (Shanghai, China). Empty lentiviral vector was used as negative control. Cell transfection was conducted with Lipofectamine RNAiMAX or Lipofectamine 3000 (Invitrogen, Carlsbad, CA, USA) according to manufacturer’s instructions. The sequences of siRNAs, miRNA mimics, miRNA inhibitor used in the study were listed in Supplementary Table 1.

### Tissue microarray and in situ hybridization (ISH)

Tissue microarray kits for human liver cancer and paired adjacent normal tissues (HlivH180Su15) were purchased from Shanghai Outdo Biotech Company (Shanghai, China). The clinicopathological features of 87 HCC patients were shown in supplementary Table 2. The study received approval from the Ethics Committee of Shanghai Outdo Biotech Company. The expression level of circ_MAPK9 in tissues was evaluated by in situ hybridization (ISH) using a specific digoxin-labeled circ_MAPK9 probe on tissue microarrays, which contained 87 tumor tissues and 86 adjacent non-tumor tissues. Briefly, the tissue microarrays were dewaxed and rehydrated, then digested with proteinase K, and then hybridized with circ_MAPK9 probe overnight at 45°C. After that, the tissues were incubated with anti-digoxin biotin conjugated antibodies overnight at 4°C and then stained with DAB. The expression of circ_MAPK9 was quantified by the scores of positive staining intensity (strong = 3, moderate = 2, weak = 1, suspicious positive = 0.5, and negative = 0) and the percentage of positive-stained cells (0%-100%). The total score is the product of “staining intensity score” and “staining positive rate”. The group with high or low expression was defined according to whether circ_MAPK9 ISH score in cytoplasm was greater than 80%. The sequence of the circ_MAPK9 probe for ISH was Digoxin-5’-CAGCTGGTATAATTCATAGGATCTGAAACTTGCCCACC-3’-Digoxin (BOSTER BIOLOGICAL CHNOLOGY, Wuhan, China).

### Western blot assay

Cells were lysed with RIPA buffer (Beyotime Biotechnology, Shanghai, China) containing protease inhibitor (Boster Biological Technology, Wuhan, China). Equal amounts of protein were separated by electrophoresis on 10% SDS-PAGE gels and transferred onto methanol-activated polyvinylidene fluoride (PVDF) membranes in 1.5 h at 90 V condition, the PVDF membranes were blocked with 5% non-fat milk for 1 h at 25 °C. Then, membranes were incubated with primary antibody for anti-poly (ADP-ribose) polymerase (PARP; 1:1,000; cat. no.9532; Cell Signaling Technology, Inc.), anti-Bcl-2 (1:1,000; cat. no.2870; Cell Signaling Technology, Inc.), MAPK9 (1:1,000; cat. no.4370; Cell Signaling Technology, Inc.), STAT3 (1:1,000; cat. no.4904; Cell Signaling Technology, Inc.), p-STAT3 (1:1,000; cat. no.4145; Cell Signaling Technology, Inc.), LDHA (1:1,000; cat. no.3582; Cell Signaling Technology, Inc.) and anti-GAPDH (1:1,000; cat. no. 2118; Cell Signaling Technology, Inc.) overnight at 4℃. The membranes were subsequently incubated with horseradish peroxidase-conjugated anti-rabbit or anti-mouse IgG secondary antibody (1:5,000; cat. no. 7074 or 7076; Cell Signaling Technology, Inc.) for 1 h at 25 °C. The protein bands were detected by enhanced chemiluminescence (SuperSignal West Pico Chemiluminescent Substrate; Pierce; Thermo Fisher Scientific, Inc.). ImageJ software (National Institutes of Health, Bethesda, MD, United States) was used to scan and analyze the protein bands.

### Real-time PCR validation

The total RNA was synthesized into cDNA with PrimeScript RT Reagent Kit (Takara Biotechnology, Tokyo, Japan) in accordance with the manufacturer’s protocols. The cDNA was amplified with TB Green Premix Ex Taq (Takara) on ABI Prism 7,300 realtime PCR system (Applied Biosystems, Foster City, CA,United States). The expression of circ_MAPK9, miR-642b-3p and mRNA were determined by 2^–ΔΔCT^ and normalized by GAPDH. The primers used in the study were listed in Supplementary Table 3.

### Luciferase reporter assay

HEK293T cells (5 × 10^4^) were seeded in 24-well plates overnight. Subsequently, 120ng pmirGLO-circ_MAPK9-WT, pmirGLO-STAT3-WT or pmirGLO-LDHA-WT reporter plasmids and their respective mutated vectors were co-transfected into cells with 50nM miR-642b-3p mimics by Lipofectamine 3000. After 48 h of cells culture, Firefly and Renilla luciferase activities were measured using the dual luciferase Reporting Detection System (Promega, Madison, Wisconsin, USA), according to the manufacturer’s instructions. The relative luciferase activities were calculated based on Firefly/Renilla fluorescence.

### Cell counting Kit-8 (CCK8) assay, EdU incorporation assay and apoptosis assay

The HCC cell viability was performed using CCK8 assay (bimake, USA). The Cell-Light™ EdU Apollo®567 In Vitro Imaging Kit (RiboBio, Guangzhou, China) was used to evaluate cell proliferation. The Annexin V-FITC/PI Apoptosis Detection Kit (Thermo Fisher, USA) was employed to assess the level of cellular apoptosis.

### Transwell migration and Boyden chamber invasion assays

HCC cells were transfected with siRNAs and incubated for 24 h. For transwell migration assay, 1 × 10^5^ SMMC-7721 or 5 × 10^4^ SK-Hep1 cells suspended in 200 µl serum-free DMEM media were seeded into the upper chamber of Transwell (Corning). DMEM containing 10% FBS (600 µl) was added into the bottom chamber. SMMC-7721 cells were incubated for 24 h at 37℃, and SK-Hep1 for 8 h. Subsequently, the filters were washed twice with PBS. Before staining with crystal violet, cells adhered on the lower surface of filters were fixed with 100% methanol at room temperature. The cell numbers in each well were microscopically counted on 5 random areas (OLYMPUS; magnification: × 200). The procedure for Boyden chamber invasion assay was similar to that of transwell migration assay except additional precoated transwell membrane with matrigel (BD Biosciences, San Jose, CA).

### In vivo assay

Male BALB/c nude mice (4 weeks old, n = 6) were randomly divided into three groups. Vechchle, sh-circ_MAPK9, and sh-NC SMMC-7721 cells (1 × 10^7^) were subcutaneously injected into the underarm region,accordingly. After five weeks, the mice were sacrificed and the xenografts were removed. The tumor size of nude mice was measured by a caliper and calculated according to the formula (width^2^ × length)/2. In vivo experiments were performed in accordance with the guidelines for the use of laboratory animals and approved by the Animal Ethics Committee of the Second Affiliated Hospital of Guangzhou Medical University.

### Statistical analysis

Statistical analyses were conducted by SPSS 16.0 software (SPSS Inc. Chicago, IL, United States). The data was shown as mean ± SD of triplicates. *P* values were calculated using Student’s *t*-test or one-way ANOVA. *P* < 0.05 indicated a significant difference. Survival analysis was performed on GraphPad Prism 5 and Kaplan-Meier curves and log-rank test were used for significance analysis.

## Results

### Circ_MAPK9 is up-regulated in HCC cells and human liver cancer tissues

**Fig. 1:**
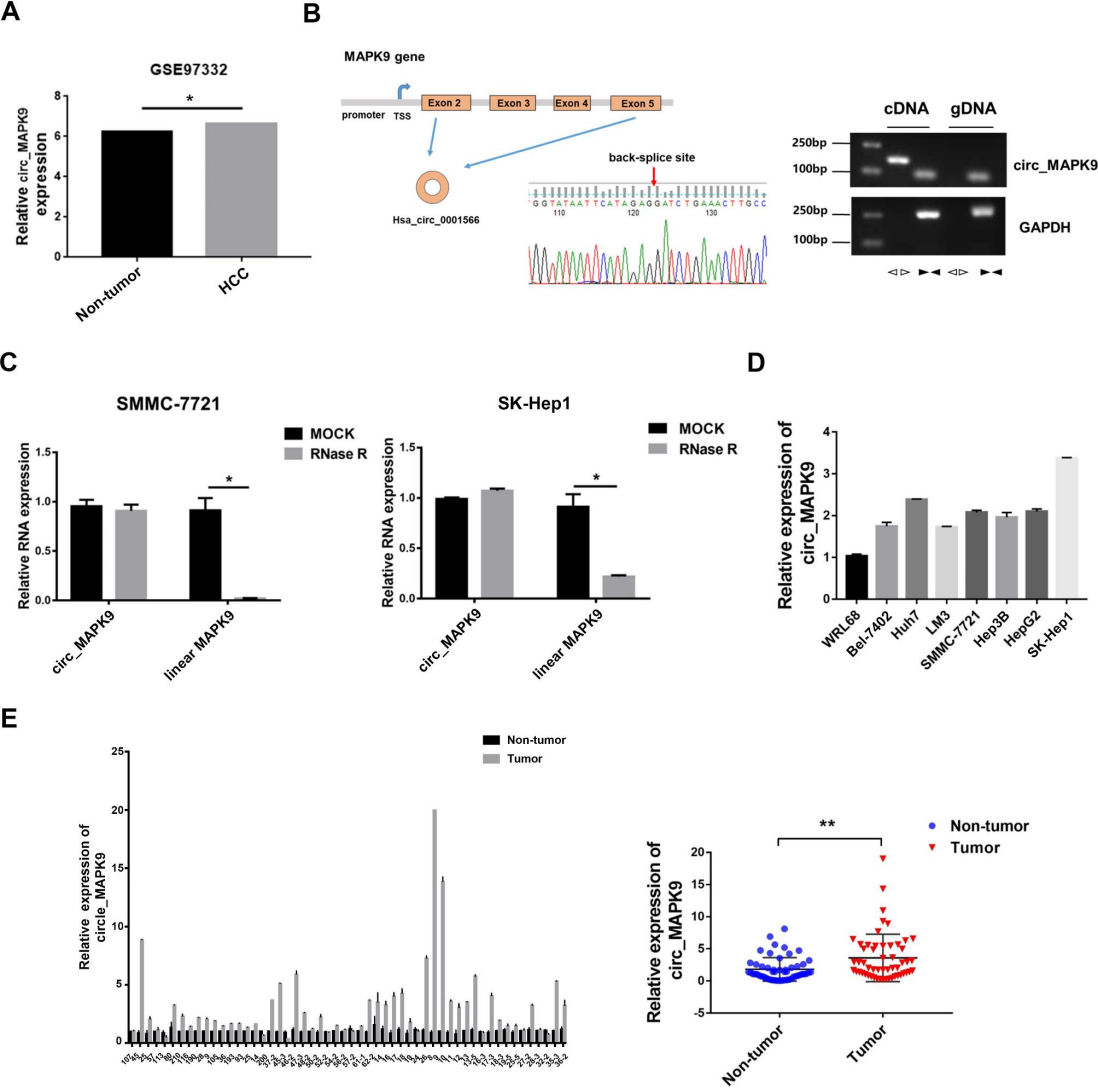
Identification of the circular structure of circ_MAPK9 and its expression in HCC cells and liver cancer tissues. **A**, The expression level of circ_MAPK9 in HCC patient data from GSE97332 dataset. **B**, The existence of circ_MAPK9 was validated in SMMC-7721 cell line by RT-PCR. Convergent primer amplified circ_MAPK9 fragments from cDNA and gDNA. Divergent primers amplified circ_MAPK9 fragment from cDNA, but not from gDNA. GAPDH was used as a negative control. The red arrow represented the “head-to-tail” splicing site of circ_MAPK9 according to Sanger sequencing. **C**, The expression level of circ_MAPK9 and linear MAPK9 mRNA in SMMC-7721 and SK-Hep1 cells treated with RNase R, which were normalized to mock treatment, determined by qRT-PCR. **D**, The expression levels of circ_MAPK9 in normal hepatocytes WRL68 and HCC cell lines. **E**, Circ_MAPK9 expression in 54 paired clinical HCC tissues, detected by qRT-PCR. **P < 0.01

To identify circRNAs in HCC tissues, we analyzed the gene expression data series (GSE) dataset GSE97332 (HCC patient data) and revealed that circ_MAPK9 was up-regulated in HCC tissue (Fig. 1A). Circ_MAPK9 consisted of 4 exons (exons 2–5) from MAPK9 genome (Fig. 1B), and we validate the circular structure of circ_MAPK9 using several universal circRNA detection methods. First, we designed convergent primers to amplify MAPK9 mRNA and divergent primers to amplify circ_MAPK9, respectively. Using cDNA and genomic DNA (gDNA) from SMMC-7721 cells as templates, circ_MAPK9 was amplified only from cDNA by divergent primers, while no amplification was observed from gDNA. Then, Sanger sequencing confirmed the head-to-tail splicing in circ_MAPK9 RT-PCR product with the expected size (Fig. 1B). Furthermore, the circularization of circ_MAPK9 was confirmed by treating RNAs extracted from HCC cells with or without RNAse R. The results showed that RNAse R reduced the linear mRNA level of MAPK9, not affected circ_MAPK9 (Fig. 1C). Our studies also displayed that HCC cell lines expressed the higher circ_MAPK9 levels compared to those in normal hepatocytes WRL68 (Fig. 1D) and similar phenomena were observed in HCC tissues and adjacent normal liver tissues (Fig. 1E).

### Circ_MAPK9 promotes the proliferation, migration and invasion of HCC cells, but inhibits apoptosis

**Fig. 2:**
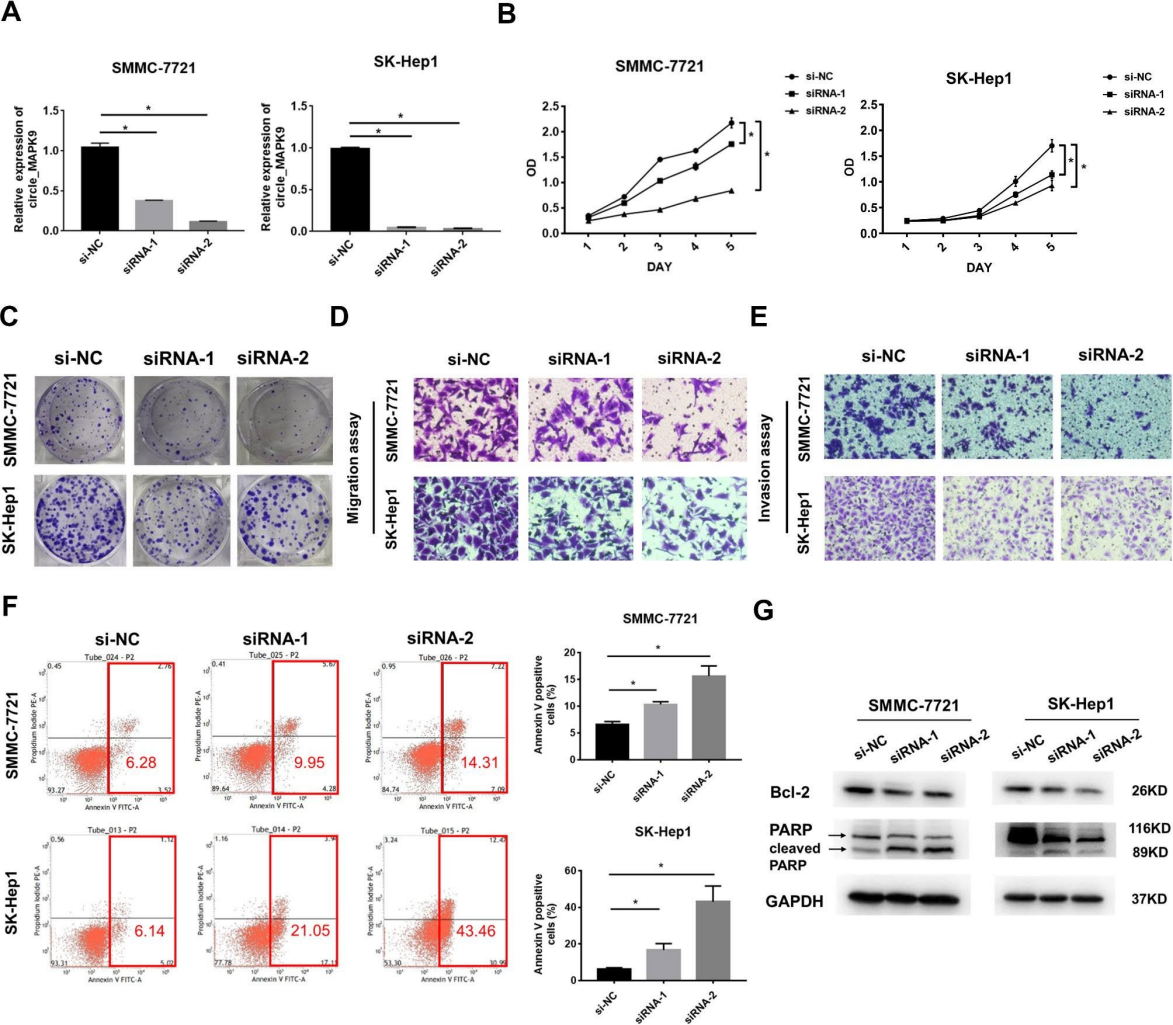
Knockdown of circ_MAPK9 inhibits the proliferation, migration and invasion of HCC cells, but induces cellular apoptosis. **A**, The knockdown efficiency of circ_MAPK9 in SMMC-7721 and SK-Hep1 cells, determined by qRT-PCR. **B–C**, Circ_MAPK9 knockdown significantly reduced the proliferative capacity of SMMC-7721 and SK-Hep1 cells, determined by CCK8 and colony formation experiments. **D-E**, Circ_MAPK9 knockdown suppressed HCC cells migration and invasion, evaluated by the transwell and Boyden assay (magnification: ×200). **F**, Cells with silenced circ_MAPK9 expression were stained with a combination of Annexin V and PI and analyzed by Flow cytometric assay (FACS). Cells positive for Annexin V staining were counted as apoptotic cells, and the percentage of apoptotic cells is shown. **G**, The expression of apoptosis related proteins, measured by Western blotting assay. GAPDH was used as a loading control (*P < 0.05)

To investigate the biological function of circ_MAPK9 in the development and progression of HCC, we used siRNAs to knock down circ_MAPK9 expression in SMMC-7721 and SK-Hep1 cells. As shown in Fig. 2A, the knockdown of circ_MAPK9 was successfully achieved using circ_MAPK9 specific siRNA-1 and siRNA-2. Cell Counting Kit-8 (CCK-8) and colony formation assays revealed that knockdown circ_MAPK9 expression inhibited proliferation of HCC cells (Fig. 2B-C). Our transwell migration assay indicated that HCC cells with circ_MAPK9 knockdown exhibited the decreased migration ability (Fig. 2D, S. Figure 1 A). Meanwhile, the Boyden chamber invasion assays showed that the invasion ability of circ_MAPK9 silenced HCC cells was impaired (Fig. 2E, S. Figure 1B). We also observed that the percentage of Annexin V positive cells in circ_MAPK9 knockdown HCC cells was significantly larger than that in the control group (Fig. 2F). Circ_MAPK9 knockdown down-regulated Bcl-2 protein expression in HCC cells, and up-regulated the cleaved PARP protein expression (Fig. 2G, S. Figure 2). These results indicate that down-regulated circ_MAPK9 expression can significantly inhibit the proliferation, migration and invasion of HCC cells, and promote cell apoptosis.

### Increased circ_MAPK9 expression is associated with poor survival patients with HCC

**Fig. 3:**
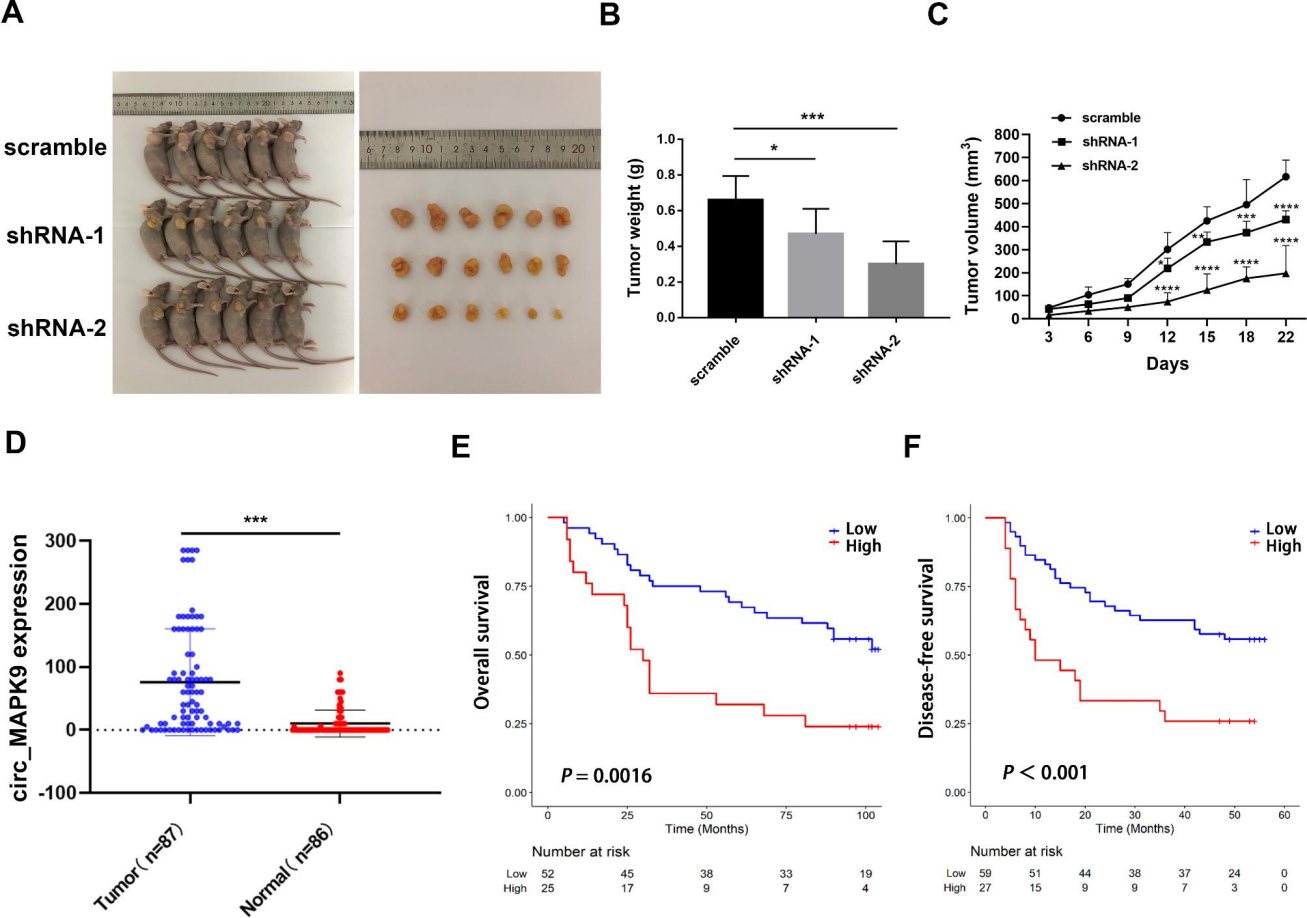
The correlation between circ_MAPK9 expression and HCC patients’ survival. **A**, The effect of circ_MAPK9 knockdown on tumor growth in vivo. Representative images of tumors formed in nude mice by subcutaneous injection of circ_MAPK9-silenced SMMC-7721 cells. **B-C**, The weight and volume of xenograft tumors displayed as mean ± SD in scramble and circ_MAPK9-silenced groups. **D**, The expression level of circ_MAPK9 in human liver cancer and paired adjacent normal tissues. **E-F**, Kaplan-Meier analysis of OS and DFS rate in HCC patients based on circ_MAPK9 expression level (*P < 0.05, **P < 0.01, ***P < 0.001, ****P < 0.001 vs. scramble group)

In this study, we established the tumor model by subcutaneously transplanting SMMC-7721 cells into nude mice. The results displayed that the growth of xenograft tumors from circ_MAPK9 knockdown cells was slower and tumor size was smaller compared to tumors grown from the corresponding control cells (Fig. 3A-C). These evidence further demonstrated that circ_MAPK9 posessed the oncogenic effect. We analyzed the correlation between circ_MAPK9 and clinicopathological status of HCC patients to determine whether circ_MAPK9 expression level was associated with HCC progression. The results showed that circ_MAPK9 was overexpressed in HCC tissues relative to the corresponding adjacent normal liver tissues (*P* < 0.001, Fig. 3D). Although no significant difference was observed between circ_MAPK9 and gender, age, pathological grade, tumor size, TNM stage and other clinicopathologic characteristics (Supplementary Table 4), Kaplan–Meier and log-rank test analyses suggested that the higher circ_MAPK9 expression in patients with HCC was negatively correlated with overall survival (OS) and disease-free survival (DFS) of HCC patients (*P* = 0.0016 for OS, *P* < 0.001 for DFS; Fig. 3E-F). Univariate analysis showed that circ_MAPK9 expression (*P* = 0.003) and tumor size (*P* = 0.038) were significantly correlated with OS rate in HCC patients (Table 1). The multivariate analysis revealed that circ_MAPK9 expression was a significant and independent prognostic factor for HCC patients (95% confidence interval [CI], 1.433–4.860; *P* = 0.002) except for tumor size (95% CI, 1.081–3.721; *P* = 0.027, Table 1). Taken together, the high expression of circ_MAPK9 in HCC tissues predicts poor prognosis of HCC patients.

**Table 1:** Univariate and Multivariate Analyses of OS in 87 HCC Patients by Cox Regression Analysis

Variables	Univariate analysis				Multivariate analysis			
	*P* value	HR	95% CI		*P* value	HR	95% CI	
Circ_MAPK9 expression	0.003	2.559	1.391–4.706		0.002	2.639	1.433–4.860	
Gender	0.539	0.692	0.214–2.240					
Age	0.776	0.081	1–0.776					
Pathological grade	0.775	1.100	0.573–2.110					
Tumor size	0.038	1.922	1.038–3.559		0.027	2.006	1.081–3.721	
T stage	0.075	1.751	0.944–3.248					
TNM stage	0.075	1.751	0.944–3.248					

Abbreviations: OS, overall survival; HR, Hazard ratio; CI, confidence interval

*P* < 0.05: the values had statistically significant differences

### Circ_MAPK9 acts as a sponge and directly targets miR-642b-3p

**Fig. 4:**
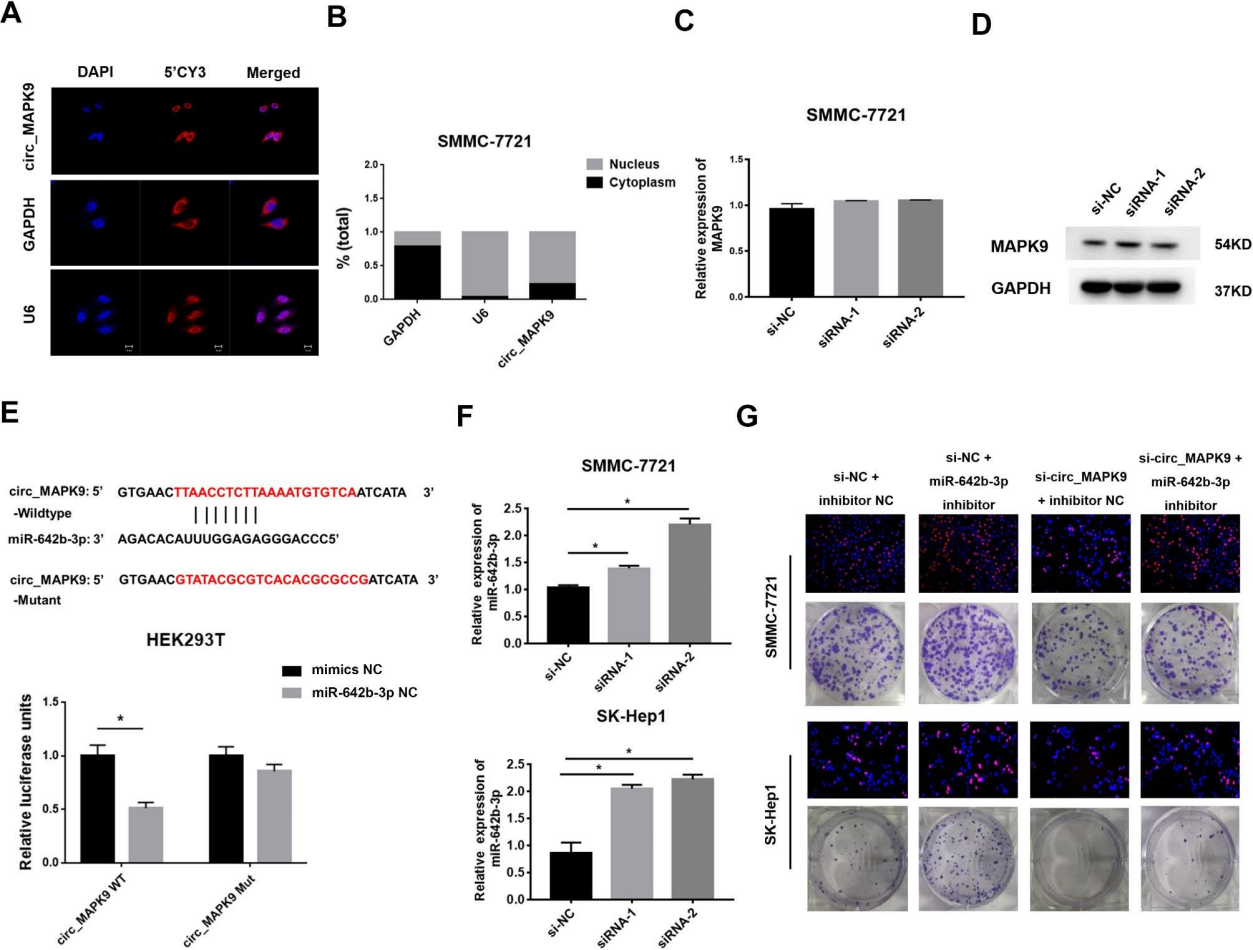
Circ_MAPK9 mediates HCC cell proliferation by regulating miR-642b-3p. **A and B**, RNA-FISH and qRT-PCR analysis of the localization of circ_MAPK9 in SMMC-7721 cells. **C and D**, The expression levels of MAPK9 mRNA and protein after down-regulated circ_MAPK9 expression, detected by qRT-PCR and Western blot. **E**, Schematic representation of the binding sites between miR-642b-3p and WT circ_MAPK9 3’UTR. Comparison of the luciferase activity of circ_MAPK9 3’UTR after treatment with miR-642b-3p mimics in HEK293T cells. **F**, The effect of circ_MAPK9 knockdown on miR-642b-3p expression in SMMC-7721 and SK-Hep1 cells analyzed by qRT-PCR. **G**, Cell proliferation after transfection with circ_MAPK9 siRNA and/or miR-642b-3p inhibitor in SMMC-7721 and SK-Hep1 cells, analyzed by EdU and colony formation assay. *P < 0.05

To investigate the regulatory mechanism of circ_MAPK9 in HCC, we detected its subcellular localization in HCC cells. RNA-FISH and subcellular fractionation assays revealed that circ_MAPK9 expressed in both nucleus and cytoplasm, but there was abundant signals and enrichment in the nucleus (Fig. 4A-B). It has been reported that the circRNAs located in the nucleus regulate parental genes or interact with transcription factors, while the circRNAs located in the cytoplasm may act as miRNA sponges [4, 12]. Examining the mRNA and protein expression of circ_MAPK9 parental gene revealed that down-regulation of circ_MAPK9 expression did not affect the expression of MAPK9 mRNA and protein level (Fig. 4C-D, S. Figure 3). Then, we attempted to identify the downstream miRNAs of circ_MAPK9 through RNA-RNA interactome in ENCORI platform and RegRNA 2.0 websites (https://rnasysu.com/encori/agoClipRNA.php?source=circRNA&flag=miRNA&clade=mammal&genome=human&assembly=hg38&miRNA=hsa-miR-642b-3p&clipNum=regionType=&pval=&clipType=&deNum=&target=), and found that miR-642b-3p had a putative binding site with circ_MAPK9. The correlation between circ_MAPK9 and miR-642b-3p was determined using a dual-luciferase reporter assay. As shown in Fig. 4E, transfection with miR-642b-3p mimic attenuated the luciferase activity of wild-type (WT) circ_MAPK9 compared with mimic control, while miR-642b-3p mimic did not affect the luciferase activity of circ_MAPK9 mutant. In addition, knockdown of circ_MAPK9 expression in HCC cells increased the expression of miR-642b-3p (Fig. 4F). Rescue experiments showed that circ_MAPK9 knockdown inhibited the proliferation of SMMC-7721 and SK-Hep1 cells. The miR-642b-3p inhibitor could attenuate this inhibitory effect (Fig. 4G). Collectively, circ_MAPK9 accelerates the proliferation of HCC cells by targeting miR-642b-3p.

### MiR-642b-3p is down-regulated in HCC, which inhibits the proliferation of HCC cells

**Fig. 5:**
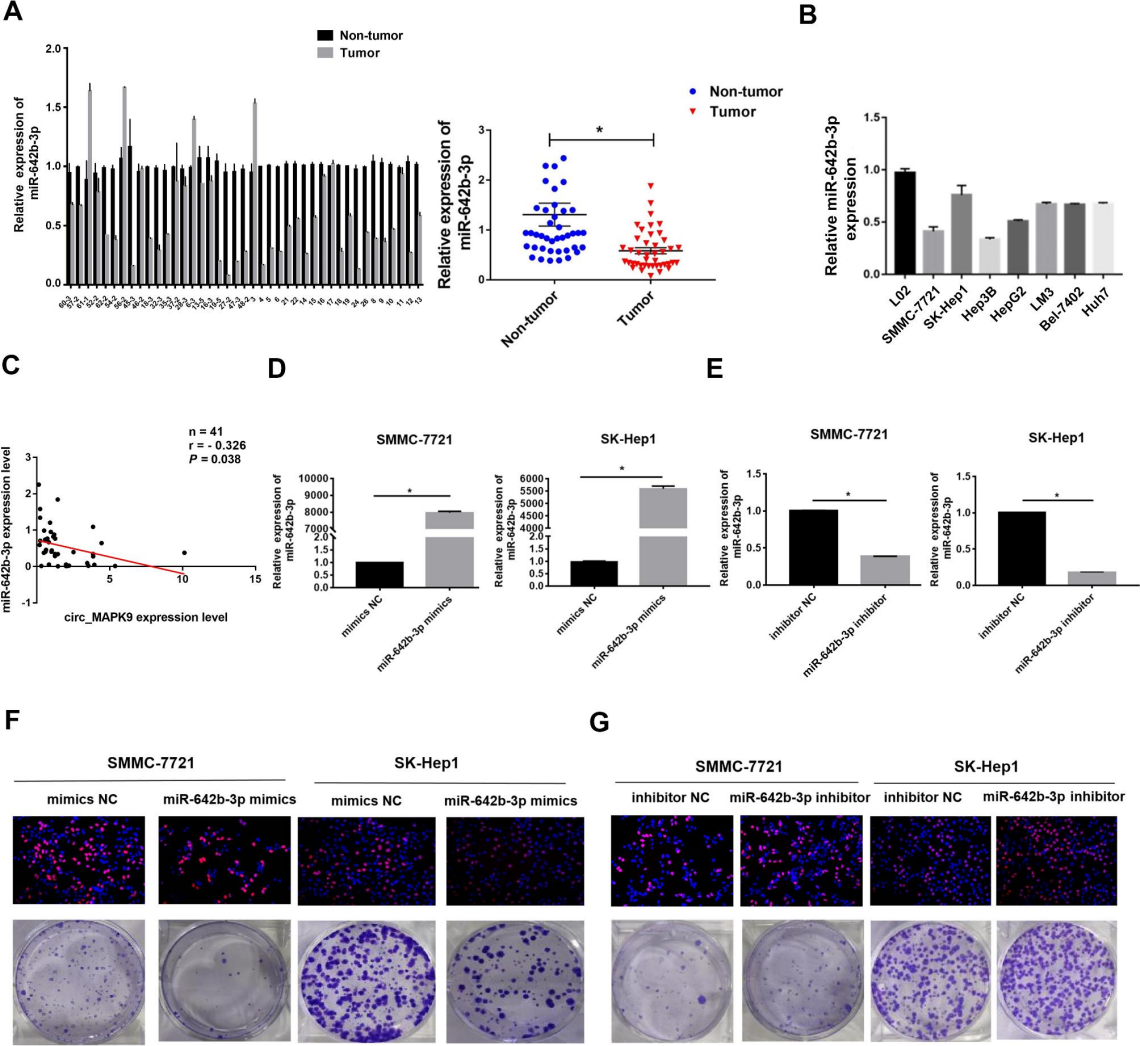
MiR-642b-3p acts as a tumor suppressor gene in HCC. **A-B**, The expression levels of miR-642b-3p in clinical HCC tissues and HCC cell lines. **C**, The expression correlation between circ_MAPK9 and miR-642b-3p in 41 pairs of HCC and corresponding non-tumor tissues was analyzed by Pearson correlation test (r = -0.326; *P* = 0.038). **D**, The overexpression efficiency of miR-642b-3p mimics in SMMC-7721 and SK-Hep1 cells detected by qRT-PCR. **E**, The knockdown efficiency of miR-642b-3p inhibitors in HCC cells detected by qRT-PCR. **F**, MiR-642b-3p overexpression significantly reduced the proliferative capacity of SMMC-7721 and SK-Hep1 cells determined by EdU and colony formation experiments. **G**, MiR-642b-3p knockdown significantly increased the proliferative capacity of SMMC-7721 and SK-Hep1 cells determined by EdU and colony formation experiments. *P < 0.05

As miR-642b-3p is the downstream target gene of circ_MAPK9, we then examined the expression of miR-642b-3p in 41 pairs of HCC samples by qRT-PCR, and found that miR-642b-3p expression decreased in HCC tumor tissues compared to that in adjacent normal tissues (*P* < 0.05, Fig. 5A). The expression of miR-642b-3p in HCC cell lines was lower than that in normal hepatocyte LO2 cell lines (Fig. 5B). We also observed a negative correlation between the expression of circ_MAPK9 and miR-642b-3p in 41 HCC samples (r = -0.326; *P =* 0.038; Fig. 5C). Additionally, miR-642b-3p mimics or inhibitors were used to overexpress or knock down the expression of miR-642b-3p in HCC cells, and the transfection efficiency was shown in Fig. 5D-E. We observed that overexpression of miR-642b-3p inhibited the proliferation of SMMC-7721 and SK-Hep1 cells (Fig. 5F). Conversely, inhibition of miR-642b-3p expression promoted cellular proliferation (Fig. 5G). These results further suggest that circ_MAPK9 promotes HCC progression by targeting miR-642b-3p.

### Circ_MAPK9 promotes the proliferation of HCC cells by regulating the miR-642b-3p/STAT3 axis

**Fig. 6:**
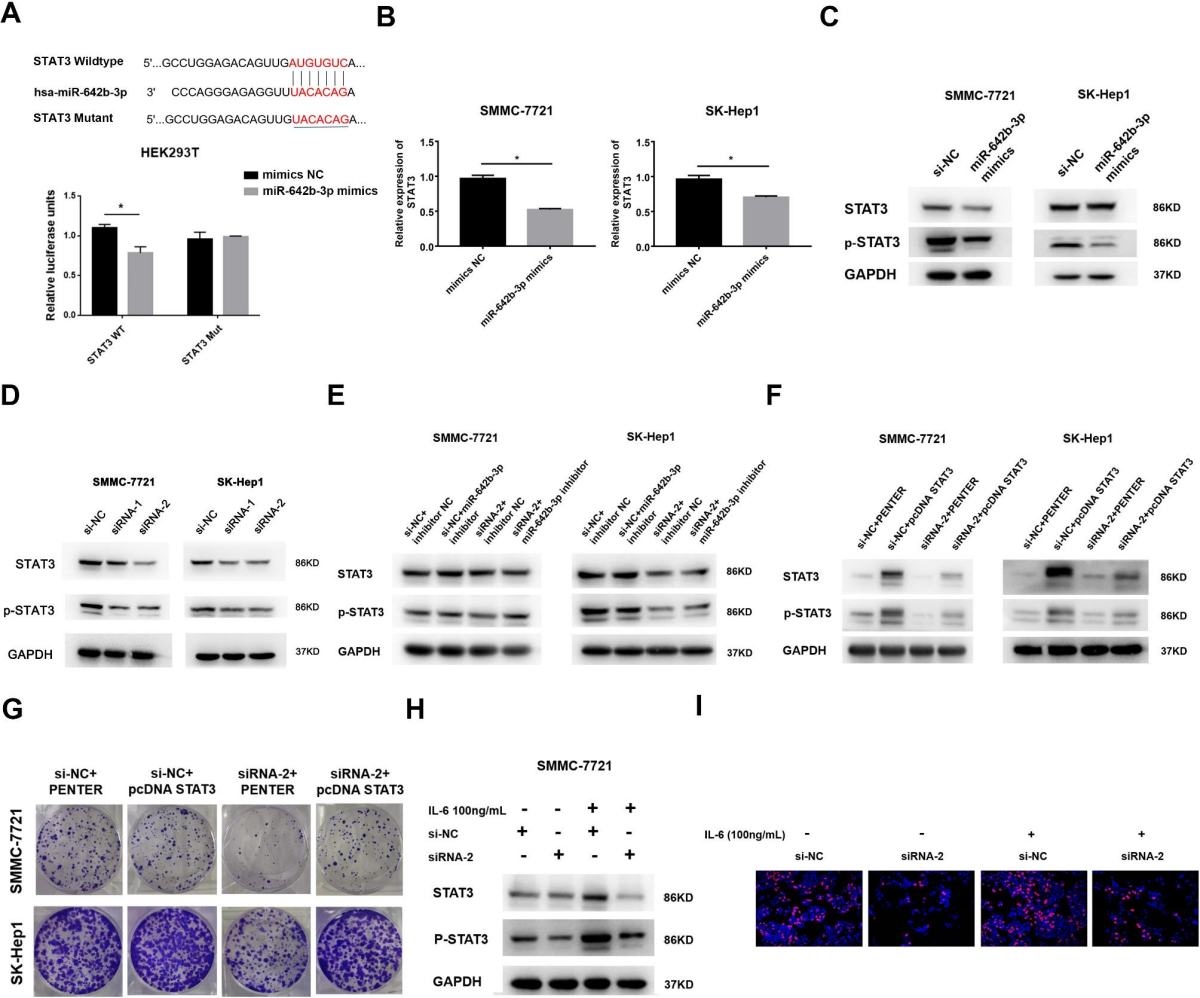
STAT3 is regulated via circ_MAPK9/miR-642b-3p axis in HCC. **A**, Schematic representation of the binding sites between miR-642b-3p and STAT3 3’UTR. Comparison of the luciferase activity of WT or MUT STAT3 3’UTR after treatment with miR-642b-3p mimics in HEK293T cells. **B and C**, The expression of STAT3 mRNA and protein in SMMC-7721 and SK-Hep1 cells transfected with miR-642b-3p mimics analyzed by qRT-PCR and Western blot. **D**, The expression of STAT3 and p-STAT3 protein in HCC cells transfected with circ_MAPK9 siRNA analyzed by Western blot. **E**, The STAT3 and p-STAT3 protein expression after transfection with circ_MAPK9 siRNA and/or miR-642b-3p inhibitor in HCC cells, analyzed by Western blot. **F**, The STAT3 and p-STAT3 protein expression after transfection with circ_MAPK9 siRNA and/or STAT3 plasmid in HCC cells, analyzed by Western blot. G, The proliferative capacity of SMMC-7721 and SK-Hep1 cells after transfection with circ_MAPK9 siRNA and/or STAT3 plasmid detected by colony formation assay. **H**, The STAT3 and p-STAT3 protein expression in circ_MAPK9 knockdown SMMC-7721 cells treated with IL-6, analyzed by Western blot. I, The proliferative capacity of circ_MAPK9 knockdown SMMC-7721 cells treated with IL-6 detected by EdU assay

Bioinformatics analysis of TargetScan (https://www.targetscan.org/cgi-bin/targetscan/vert_71/targetscan.cgi?mirg=hsa-miR-642b-3p) revealed a potential binding relationship between miR-642b-3p and STAT3. Luciferase reporter assay confirmed the molecular interaction between miR-642b-3p and STAT3 (Fig. 6A). Overexpression of miR-642b-3p in HCC cells inhibited the expression of STAT3 mRNA and protein, and decreased the level of phosphorylated STAT3 (Tyr705) expression (Fig. 6B-C, S. Figure 4A). Down-regulated circ_MAPK9 expression by siRNA reduced the expression of STAT3 and its phosphorylated protein (Fig. 6D, S. Figure 4B). To further determine whether circ_MAPK9 plays an oncogenic role in HCC via miR-642b-3p/STAT3 axis, we performed a rescue assay. Knockdown of miR-642b-3p could reverse the down-regulation of STAT3 and its phosphorylated protein expression caused by circ_MAPK9 knockdown (Fig. 6E, S. Figure 4C). In addition, the cloning experiments showed that overexpression of STAT3 could reverse the inhibitory effect caused by circ_MAPK9 knockdown on the proliferation of HCC cells (Fig. 6F-G, S. Figure 4D). We also used an agonist of STAT3 (IL-6) to confirm the relationship between circ_MAPK9 and STAT3. Western blot assay revealed that the expression of phosphorylated STAT3 protein in IL-6 added group was up-regulated, compared with circ_MAPK9 knockdown group (Fig. 6H, S. Figure 4E). Meanwhile, IL-6 accelerated the proliferation of circ_MAPK9 knockdown HCC cells (Fig. 6I). All these data suggest that STAT3 serve as downstream factor in the circ_MAPK9/miR-642b-3p axis.

### Circ_MAPK9 exacerbates HCC progression by partially regulating the miR-642b-3p/LDHA axis

**Fig. 7:**
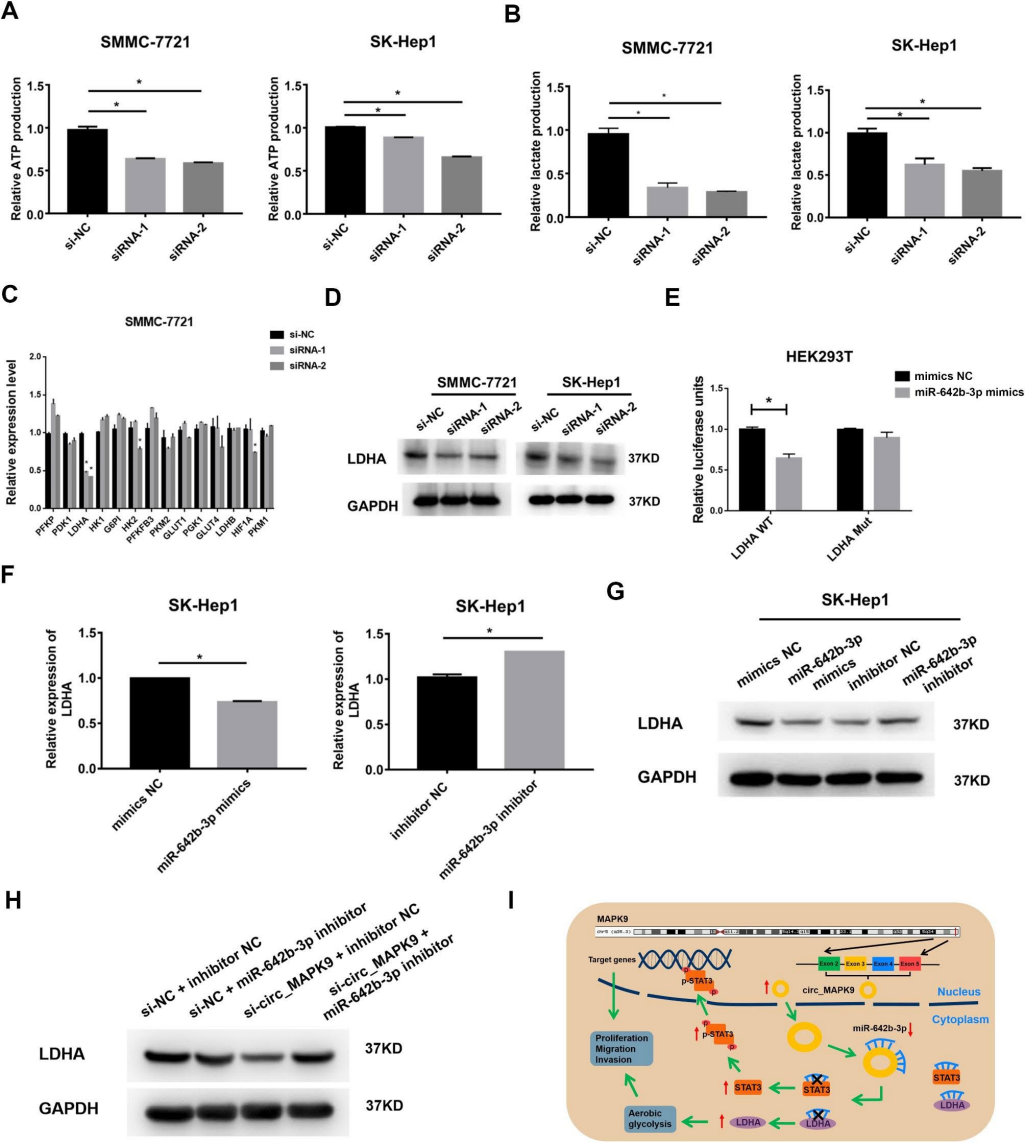
Circ_MAPK9/miR-642b-3p/LDHA axis regulates aerobic glycolysis in HCC. **A-B**, ATP and lactic acid production in circ_MAPK9 knockdown HCC cells. **C**, The effect of circ_MAPK9 knockdown on the expression of glycolytic genes, analyzed by qRT-PCR. **D**, The effect of circ_MAPK9 knockdown on LDHA protein expression, analyzed by Western blot. **E**, Comparison of the luciferase activity of WT or MUT LDHA 3’UTR after treatment with miR-642b-3p mimics in HEK293T cells. **F-G**, The expression of LDHA mRNA and protein in SK-Hep1 cells transfected with miR-642b-3p mimics or inhibitor analyzed by qRT-PCR or Western blot, respectively. **H**, LDHA protein expression after transfection with circ_MAPK9 siRNA and/or miR-642b-3p inhibitor in SK-Hep1 cells, analyzed by Western blot

Next, we explored the effect of circ_MAPK9 on aerobic glycolysis by detecting the production of adenosine triphosphate (ATP) and lactic acid. The ATP and lactate production in the circ_MAPK9 knock-downed SMMC-7721 and SK-Hep1 cells decreased compared with those in the control cells (Fig. 7A-B). To elucidate the molecular mechanism of circ_MAPK9 involved in aerobic glycolysis of HCC cells, we detected the expression of genes related to aerobic glycolysis. Circ_MAPK9 knockdown down-regulate the mRNA of lactate dehydrogenase (LDHA) and protein levels in HCC cells (Fig. 7C-D, S. Figure 5A). Coincidentally, we identified LDHA as a potential target for miR-642b-3p using TargetScan (https://www.targetscan.org/cgi-bin/targetscan/vert_71/targetscan.cgi?mirg=hsa-miR-642b-3p). The dual-luciferase reporter assays revealed that miR-642b-3p overexpression attenuated the luciferase activity of LDHA in WT, but did not affect the mutant construct (Fig. 7E). Overexpression of miR-642b-3p could decrease the expression of LDHA mRNA and protein in HCC cells, while the knockdown increased the expression of LDHA mRNA and protein as expected (Fig. 7F-G, S. Figure 5B). These data indicate that miR-642b-3p directly regulates its expression by targeting LDHA. Moreover, our experiments of simultaneous knockdown of circ_MAPK9 and miR-642b-3p revealed that miR-642b-3p knockdown could reverse the inhibitory effect of circ_MAPK9 knockdown on LDHA expression (Fig. 7H, S. Figure 5C). These findings demonstrate that the circ_MAPK9/miR-642b-3p/LDHA axis is involved in the development of HCC.

## Discussion

Our study reported that circ_MAPK9 was up-regulated in HCC and functioned as ceRNA via sponging miR-642b-3p to release the inhibition of miR-642b-3p on STAT3 and LDHA for the first time. Alleviating the suppression of miR-642b-3p on STAT3 and LDHA led to up-regulation of phosphorylated STAT3 and LDHA expression, and promoted HCC cell proliferation, invasion, and migration, in part through glycolysis (Fig. 7I). Circ_MAPK9 overexpression in HCC tissues was negatively correlated with OS and DFS of patients. Circ_MAPK9 was a significant and independent factor affecting the prognosis of patients with HCC. These results suggest that circ_MAPK9 is an oncogene in HCC and could be a potential prognostic indicator for HCC.

According to the composition and cycling mechanisms, the circRNAs can be divided into three types: exonic circRNAs, intronic circRNAs, and exon-intron circRNAs (EIciRNA) [13]. Nearly a dozen of circRNAs, including exonic and intronic circRNA, have been derived from the MAPK9 genome (http://www.circbase.org/cgi-bin/simplesearch.cgi). To date, only circRNAs cycled from exons 16–21 of MAPK9 genome have been reported in RA [11]. Circ_MAPK9 in this study is also known as hsa_circ_0001566, it is derived from exons 2–5 of MAPK9 genome (circPrimer2.0, https://www.bio-inf.cn/), which is the first report of circRNAs from these exons.

Previous reports have shown that circ_MAPK9 is up-regulated in peripheral blood mononuclear cells and fibroblast-like synoviocytes of RA patients [10, 11]. Circ_MAPK9 silencing inhibits cellular proliferation, migration, invasion, inflammatory response, and promotes apoptosis of RA-FLSs [11]. These results indicated that circ_MAPK9 acts as a pathogenic gene in RA. In this study, we observed that circ_MAPK9 was highly expressed in HCC cells and tissues. Down-regulation of circ_MAPK9 could suppress the proliferation, migration, invasion of HCC cells in vitro and in vivo, but facilitate cellular apoptosis. Our findings further confirms that circ_MAPK9 is also a pathogenic gene in HCC. Although circ_MAPK9 can be detected in PBMCs of RA patients [11], further studies are required to determine the detectability of circ_MAPK9 in PBMCs or plasma samples of individuals with HCC. There is growing evidence that abnormal expression of circRNA can predict the prognosis of patients with a variety of cancers, including HCC [14]. For example, the down-regulation of circular RNA cSMARCA5 is an independent risk factor for OS and recurrence-free survival of HCC patients after hepatectomy [15]. High expression of circPAK1 in HCC tumor tissues is associated with poor outcomes in patients with HCC [16]. Our study demonstrated that circ_MAPK9 overexpression in HCC tissues was negatively correlated with OS and DFS in HCC patients, but no statistically significant correction was found between circ_MAPK9 and the clinicopathologic characteristics. The observed outcomes could be attributed to the small sample size used in our clinical study. Hence, to better understand the role of circ_MAPK9 in HCC, a prospective, multi-center, large-sample cohort study is needed in the future.

CircRNAs exhibit diverse functionalities across multiple aspects, such as competing endogenous RNAs or miRNA sponges, interacting with RNA-binding proteins, modulating mRNAs stability and translating proteins [13]. Luo et al. found that circ_MAPK9 acts as a sponge for miR-140-3p, but the subcellular localization of circ_MAPK9 has not been resolved [11]. Studies have shown that a number of circRNAs situated in the cytoplasm display the ability to bind to miRNAs as competing RNAs, which may hinder miRNAs binding to their target mRNAs [17, 18]. On the other hand, circRNAs located in the nucleus regulate the expression of parental genes by interacting with binding sites [19, 20]. Our study demonstrated that circ_MAPK9 could be detected in both the nucleus and cytoplasm with has abundant signal and enrichment in the nucleus. Nevertheless, the expression level of parental MAPK9 gene was not affected by circ_MAPK9. The cytoplasmic function of circ_MAPK9 has been demonstrated as a competitive endogenous RNA (ceRNA) for miR-642b-3p. Silencing circ_MAPK9 inhibited the proliferation of HCC cells, and miR-642b-3p inhibitors could attenuate this inhibitory effect. This provides strong evidence that circ_MAPK9 plays an oncogenic role in HCC by regulating miR-642b-3p. To date, whether nuclear circ_MAPK9 participants in the occurrence and progression of HCC remains undetermined.

MiR-642b-3p, a 22nt microRNA, has been reported as a carcinogenic miRNA that promotes tumorigenesis and progression of gastric cancer [21]. Overexpression of miR-642b-3p suppresses cell migration and invasion, and promotes cell proliferation and apoptosis in head and neck squamous cell carcinoma [22]. Our study confirmed miR-642b-3p as a tumor suppressor gene in HCC for the first time. MiRNAs have been reported to inhibit protein translation or promote mRNA degradation by binding to complementary sites of mRNA 3’ UTR [23, 24]. We found that STAT3 and LDHA contained binding sites for miR-642b-3p using TargetScan. Dual luciferase reporter assay confirmed the direct regulatory relationship between miR-642b-3p and STAT3 or LDHA. STAT3 belongs to the signal transducer and activator of transcription (STAT) family, which regulates a variety of genes. STAT3 is inactive in unstimulated cells, but is constitutively activated in cancer cells, including HCC cells [25]. STAT3 activation requires phosphorylation of a critical tyrosine residue (Tyr705), which mediates its dimerization that is a prerequisite for nucleus entry and DNA binding. STAT3 dimers translocate to the nucleus and activate transcription of genes associated with survival, inflammation and epithelial-mesenchymal transition (EMT) [26, 27]. We observed that overexpression of STAT3 or the STAT3 agonist IL-6 reversed the inhibitory effect of circ_MAPK9 knockdown on HCC cell proliferation, confirming the modulatory relationship between circ_MAPK9, miR-642b-3p and STAT3. LDHA is one of the crucial enzymes in aerobic glycolysis that converts pyruvate to lactate [28]. Extensive literature data have confirmed that aberrant expression and activation of LDHA is closely related to liver cancer, pancreatic cancer, nasopharyngeal cancer and other cancers. In cancer cells, LDHA facilitates rapid conversion of pyruvate to lactic acid, minimizing pyruvate entry into TCA cycle in the mitochondria [29]. High levels of LDHA assist in the formation and proliferation of cancer cells by promoting EMT, angiogenesis, cytoskeletal remodeling, increased cell viability, invasion and migration [30]. Our data suggested that circ_MAPK9 affected ATP and lactate production in HCC cells. Circ_MAPK9 silencing resulted in the down-regulation of LDHA mRNA and protein levels, while miR-642b-3p silencing could reverse this inhibition of circ_MAPK9 silencing on LDHA expression. These findings highlight that circ_MAPK9/miR-642b-3p/LDHA axis is a novel mechanism to promote the proliferation of HCC cells.

## Conclusion

In summary, our study illustrates that circ_MAPK9 acts as an oncogene to facilitate HCC cell proliferation, invasion and migration, and inhibits apoptosis by regulating miR-642b-3p/STAT3 and LDHA axis. These results indicate that circ_MAPK9 will be a novel prognostic biomarker for patients with HCC, contributing to early diagnosis or improving therapeutic strategies.

### Electronic supplementary material

Below is the link to the electronic supplementary material.

Supplementary Material 1

Supplementary Material 2

Supplementary Material 3

Supplementary Material 4

Supplementary Material 5

Supplementary Material 6

Supplementary Material 7

Supplementary Material 8

Supplementary Material 9

Supplementary Material 10

## Data Availability

All data generated from this study were included in the text and supplementary material. Further enquiries can be directed to the corresponding author.
