# Supplementary figures and images for: Circ_MAPK9 promotes STAT3 and LDHA expression by silencing miR-642b-3p and affects the progression of hepatocellular carcinoma
Source: Biol Direct. 2024 Jan 2;19:4. doi: 10.1186/s13062-023-00442-1 (PMC10759731; doi:10.1186/s13062-023-00442-1)

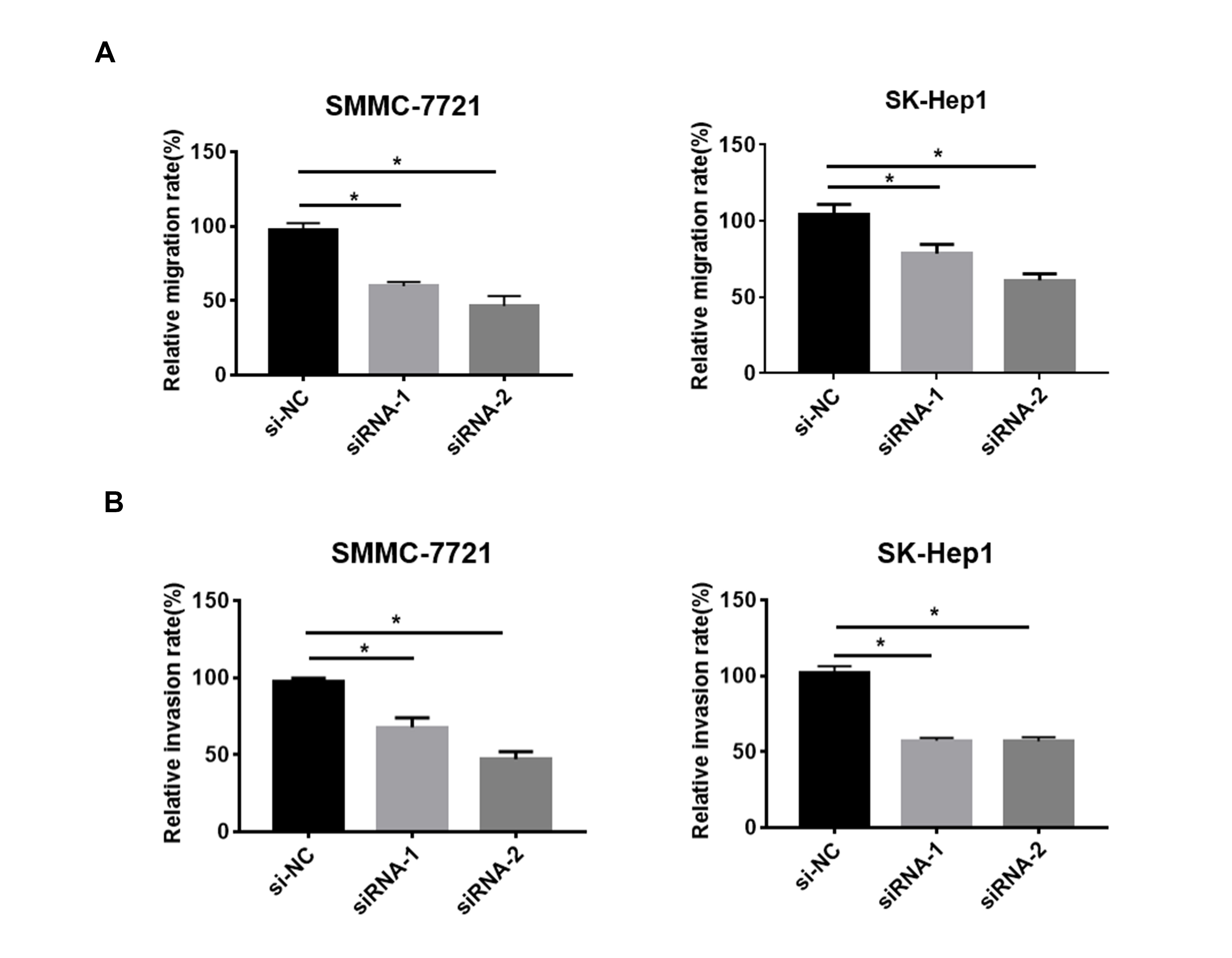

Supplement: Supplementary file 1 — Supplementary Material 1 [file 13062_2023_442_MOESM1_ESM.jpg]

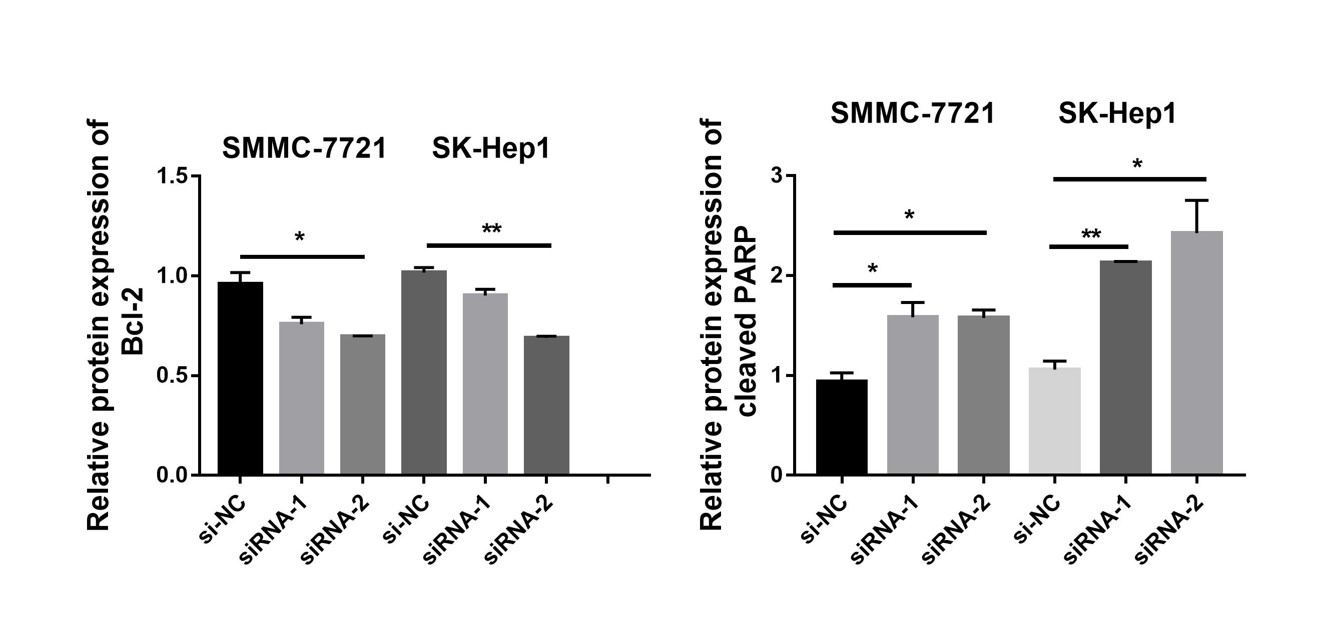

Supplement: Supplementary file 2 — Supplementary Material 2 [file 13062_2023_442_MOESM2_ESM.jpg]

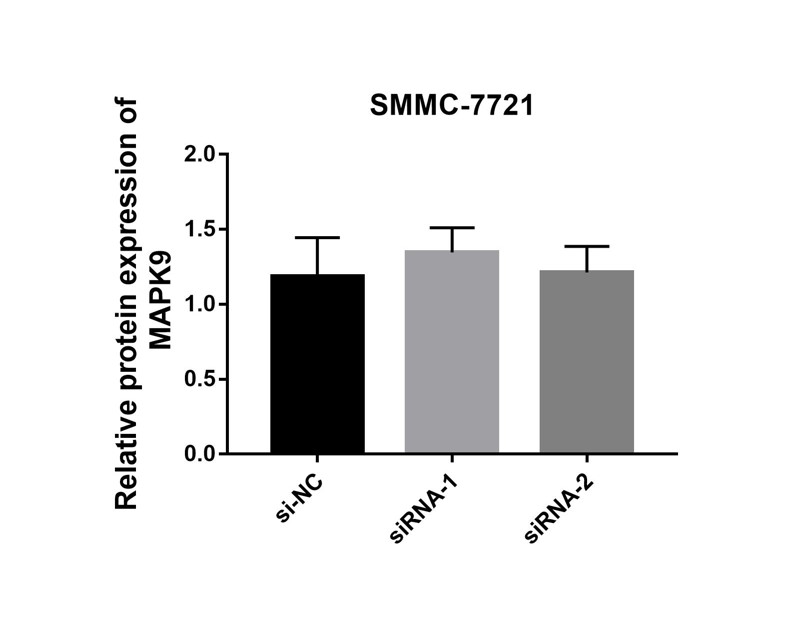

Supplement: Supplementary file 3 — Supplementary Material 3 [file 13062_2023_442_MOESM3_ESM.jpg]

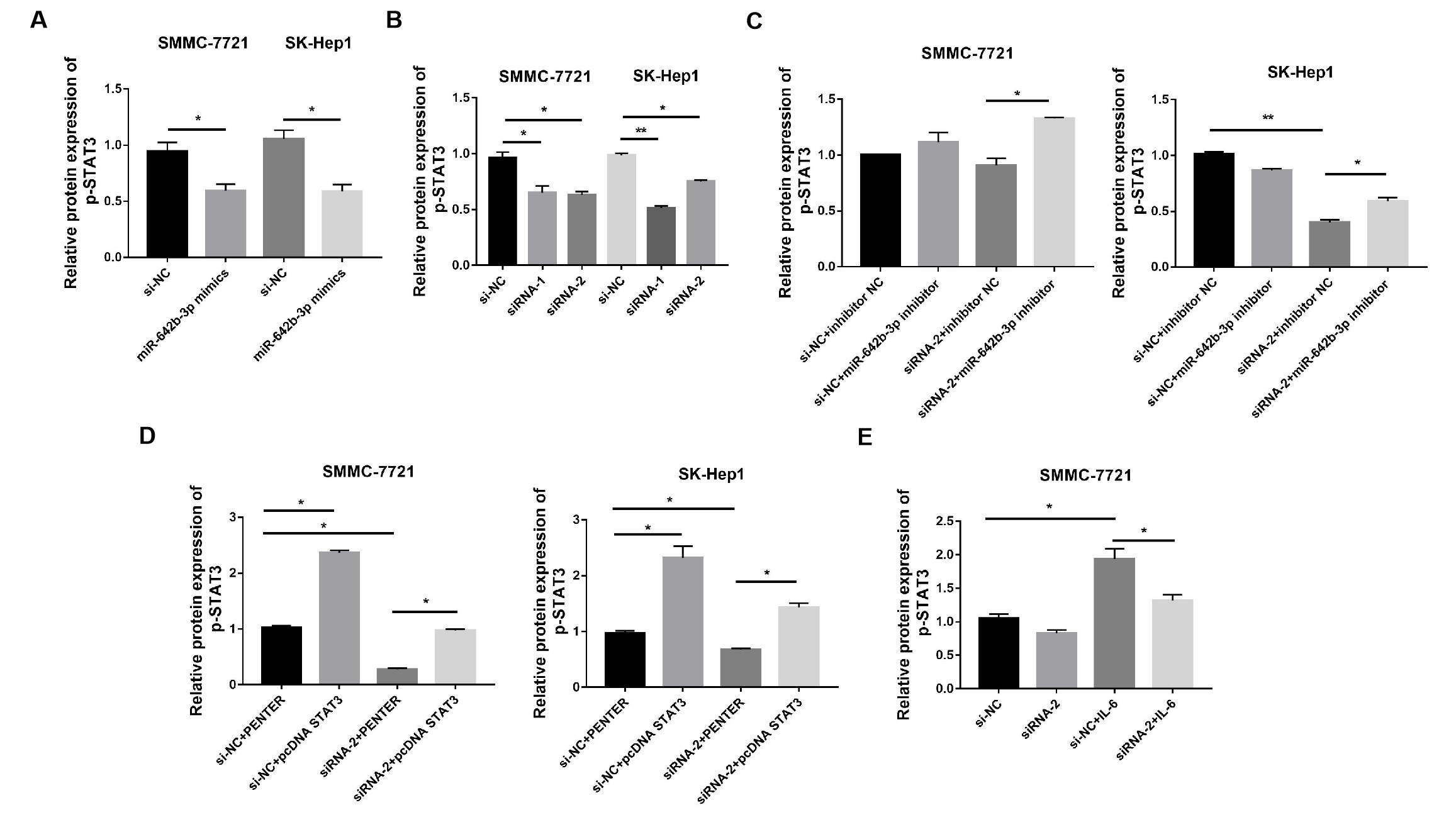

Supplement: Supplementary file 4 — Supplementary Material 4 [file 13062_2023_442_MOESM4_ESM.jpg]

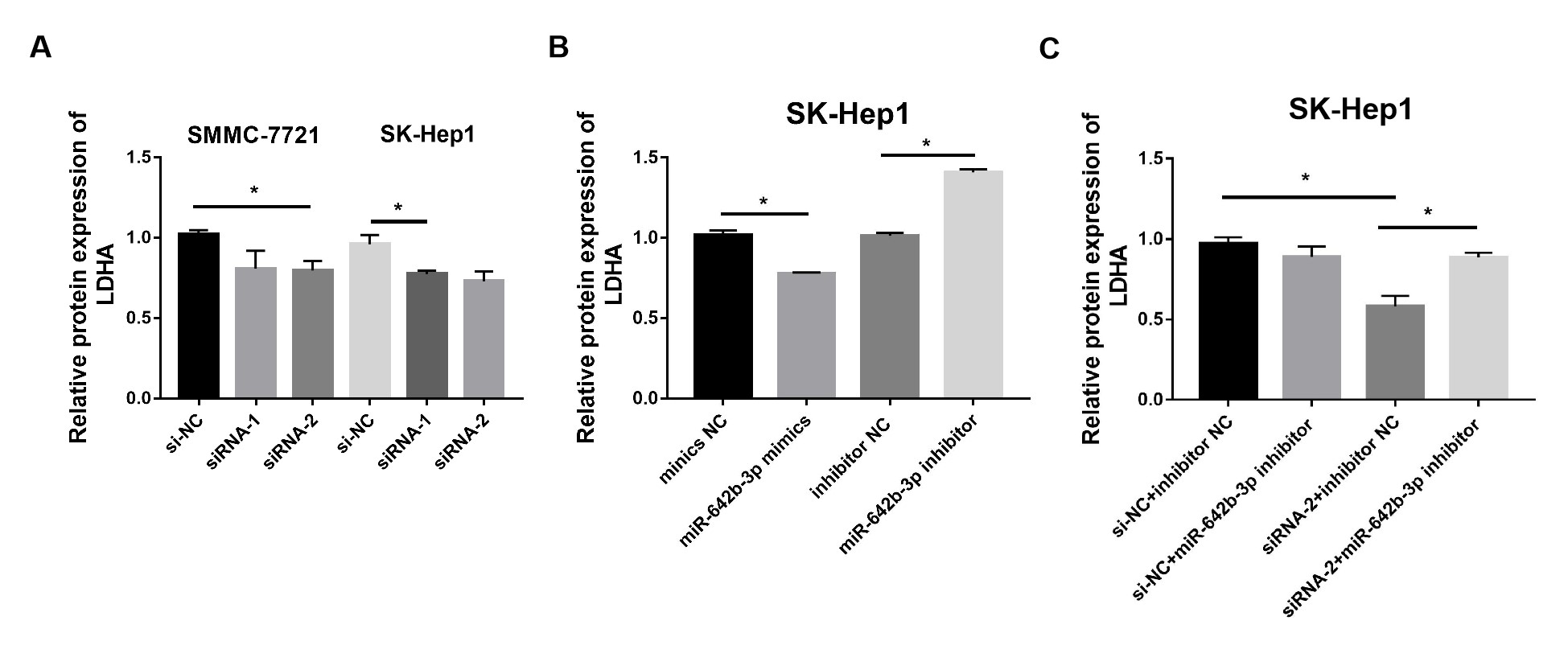

Supplement: Supplementary file 9 — Supplementary Material 9 [file 13062_2023_442_MOESM9_ESM.jpg]
