# Supplementary material for: Circ_MAPK9 promotes STAT3 and LDHA expression by silencing miR-642b-3p and affects the progression of hepatocellular carcinoma
Source: Biol Direct. 2024 Jan 2;19:4. doi: 10.1186/s13062-023-00442-1 (PMC10759731; doi:10.1186/s13062-023-00442-1)
Supplement: Supplementary file 5 — Supplementary Material 5 [file 13062_2023_442_MOESM5_ESM.doc]

**Supplementary Table 1.**

**SiRNA, miRNA mimics and inhibitor**

Primers/Probes Sequence

circ_MAPK9 siRNA-1 sense（5'-3'） GUAUAAUUCAUAGAGGAUCTT

circ_MAPK9 siRNA-1 antisense（5'-3'） GAUCCUCUAUGAAUUAUACTT

circ_MAPK9 siRNA-2 sense（5'-3'） CAUAGAGGAUCUGAAACUUTT

circ_MAPK9 siRNA-2 antisense（5'-3'） AAGUUUCAGAUCCUCUAUGTT

si-NC sense（5'-3'） UUCUCCGAACGUGUCACGUTT

si-NC antisense（5'-3'） ACGUGACACGUUCGGAGAATT

miR-642b-3p mimics sense（5'-3'） AGACACAUUUGGAGAGGGACCC

miR-642b-3p mimics antisense（5'-3'） GUCCCUCUCCAAAUGUGUCUUU

miR-642b-3p inhibitor sense（5'-3'） GGGUCCCUCUCCAAAUGUGUCU

inhibitor NC sense（5'-3'） CAGUACUUUUGUGUAGUACAA
