# Supplementary material for: Circ_MAPK9 promotes STAT3 and LDHA expression by silencing miR-642b-3p and affects the progression of hepatocellular carcinoma
Source: Biol Direct. 2024 Jan 2;19:4. doi: 10.1186/s13062-023-00442-1 (PMC10759731; doi:10.1186/s13062-023-00442-1)
Supplement: Supplementary file 7 — Supplementary Material 7 [file 13062_2023_442_MOESM7_ESM.doc]

**Supplementary Table 3.**

**Primers used for RT-qPCR**

Primers/Probes Sequence

circ-MAPK9 FP CAAGATGTGTATTTGGTTATGGA

circ-MAPK9 RP GGCAAGTTTCAGATCCTCTATG

MiR-642b-3p FP ACACTCCAGCTGGGAGACACATTTGGAGAG

MiR-642b-3p RT CTCAACTGGTGTCGTGGAGTCGGCAATTCAGTTGA GGGGTCCCT

STAT3 FP CGCACTTTAGATTCATTGATGC

STAT3 RP AGGTGAGGGACTCAAACTG
