# Supplementary material for: Circ_MAPK9 promotes STAT3 and LDHA expression by silencing miR-642b-3p and affects the progression of hepatocellular carcinoma
Source: Biol Direct. 2024 Jan 2;19:4. doi: 10.1186/s13062-023-00442-1 (PMC10759731; doi:10.1186/s13062-023-00442-1)
Supplement: Supplementary file 8 — Supplementary Material 8 [file 13062_2023_442_MOESM8_ESM.doc]

Supplementary Table 4. Correlation between circ_MAPK9 expression and HCC clinicopathological characteristics in 87 patients.

|  | variables | circ_MAPK9  expression | | total | χ^2^ | *P* value |
| --- | --- | --- | --- | --- | --- | --- |
|  |  | low | high |  |  |  |
| Gender |  |  |  |  | 0.643 | 0.423 |
|  | Female | 52 | 25 | 77 |  |  |
|  | male | 8 | 2 | 10 |  |  |
| Age (year) |  |  |  |  | 0.483 | 0.487 |
|  | ≤52 | 33 | 17 | 50 |  |  |
|  | ＞52 | 27 | 10 | 37 |  |  |
| Pathological grade |  |  |  |  | 0.005 | 0.942 |
|  | I/II | 44 | 20 | 64 |  |  |
|  | III | 16 | 7 | 23 |  |  |
| [Tumor](../../../../D:/Program%20Files%20(x86)/Youdao/Dict/8.9.6.0/resultui/html/index.html#/javascript:;) [size](../../../../D:/Program%20Files%20(x86)/Youdao/Dict/8.9.6.0/resultui/html/index.html#/javascript:;) |  |  |  |  | 0.036 | 0.849 |
|  | <5cm | 41 | 19 | 60 |  |  |
|  | ≥5cm | 19 | 8 | 27 |  |  |
| T stage |  |  |  |  | 0.097 | 0.756 |
|  | T1 | 42 | 18 | 60 |  |  |
|  | T2/T3 | 18 | 9 | 27 |  |  |
| TNM stage |  |  |  |  | 0.097 | 0.756 |
|  | I | 42 | 18 | 60 |  |  |
|  | II | 18 | 9 | 27 |  |  |

*P* < 0.05: the values had statistically significant differences.
